# A quantitative comparison between the mHand Adapt passive adjustable hand prosthesis and its predecessor, the Delft Self-Grasping Hand

**DOI:** 10.1371/journal.pone.0300469

**Published:** 2024-03-21

**Authors:** Spyros L. L. Krinis, Alix Chadwell, Laurence Kenney, Gerwin Smit

**Affiliations:** 1 Centre for Health Sciences Research, University of Salford, Salford, United Kingdom; 2 Department of Biomedical Engineering, Delft University of Technology, Delft, The Netherlands; Polytechnic University of Marche: Universita Politecnica delle Marche, ITALY

## Abstract

**Introduction:**

The Delft Self-Grasping Hand (SGH) is an adjustable passive hand prosthesis that relies on wrist flexion to adjust the aperture of its grasp. The mechanism requires engagement of the contralateral hand meaning that hand is not available for other tasks. A commercialised version of this prosthesis, known as the mHand Adapt, includes a new release mechanism, which avoids the need to press a release button, and changes to the hand shape. This study is the first of its kind to compare two passive adjustable hand prostheses on the basis of quantitative scoring and contralateral hand involvement.

**Methods:**

10 anatomically intact participants were asked to perform the Southampton Hand Assessment Procedure (SHAP) with the mHand. Functionality and contralateral hand involvement were recorded and compared against SGH data originating from a previous trial involving a nearly identical testing regime.

**Results:**

mHand exhibited higher functionality scores and less contralateral hand interaction time, especially during release-aiding interactions. Additionally, a wider range of tasks could be completed using the mHand than the SGH.

**Discussion:**

Geometric changes make the mHand more capable of manipulating smaller objects. The altered locking mechanism means some tasks can be performed without any contralateral hand involvement and a higher number of tasks do not require contralateral involvement when releasing. Some participants struggled with achieving a good initial grip due to the inability to tighten the grasp once already formed.

**Conclusion:**

The mHand offers the user higher functionality scores with less contralateral hand interaction time and the ability to perform a wider range of tasks. However, there are some design trade-offs which may make it slightly harder to learn to use.

## Introduction

Passive adjustable hand prostheses aim to have a lifelike appearance whilst still offering functionality beyond that of a static hand prosthesis [1]. This can come in the form of a grasping mechanism or some other form of adjustment. The mHand Adapt (Moveable, Ede, The Netherlands) (mHand) is a new passive adjustable hand prosthesis that utilises the extension of the prosthetic wrist to close and open the hand [2, 3]. This mechanism incorporates a spring-loaded ratchet that locks the aperture of the hand at discrete increments as the wrist is extended. To unlock the ratchet the user must slightly extend the wrist again until it clicks, indicating the mechanism has been released. Once unlocked, the springs flex the wrist until the prosthesis assumes a neutral wrist position. The opening and closing of the aperture is accomplished predominantly through flexion of the metacarpophalangeal joints of the four fingers and the proximal interphalangeal joints of all fingers but the little finger which does not have this joint. Additionally, these finger joints have some elasticity allowing the joints to shift independently to better conform the fingers to the shape of the object they are grasping. The distal interphalangeal joints of the fingers are fixed into a slight curve and are not flexible. The thumb moves with the palm of the mHand relative to the wrist and is only capable of adduction and abduction with no flexibility in any phalangeal joints. This adduction/abduction joint uses friction to hold the thumb’s position.

**Fig 1:**
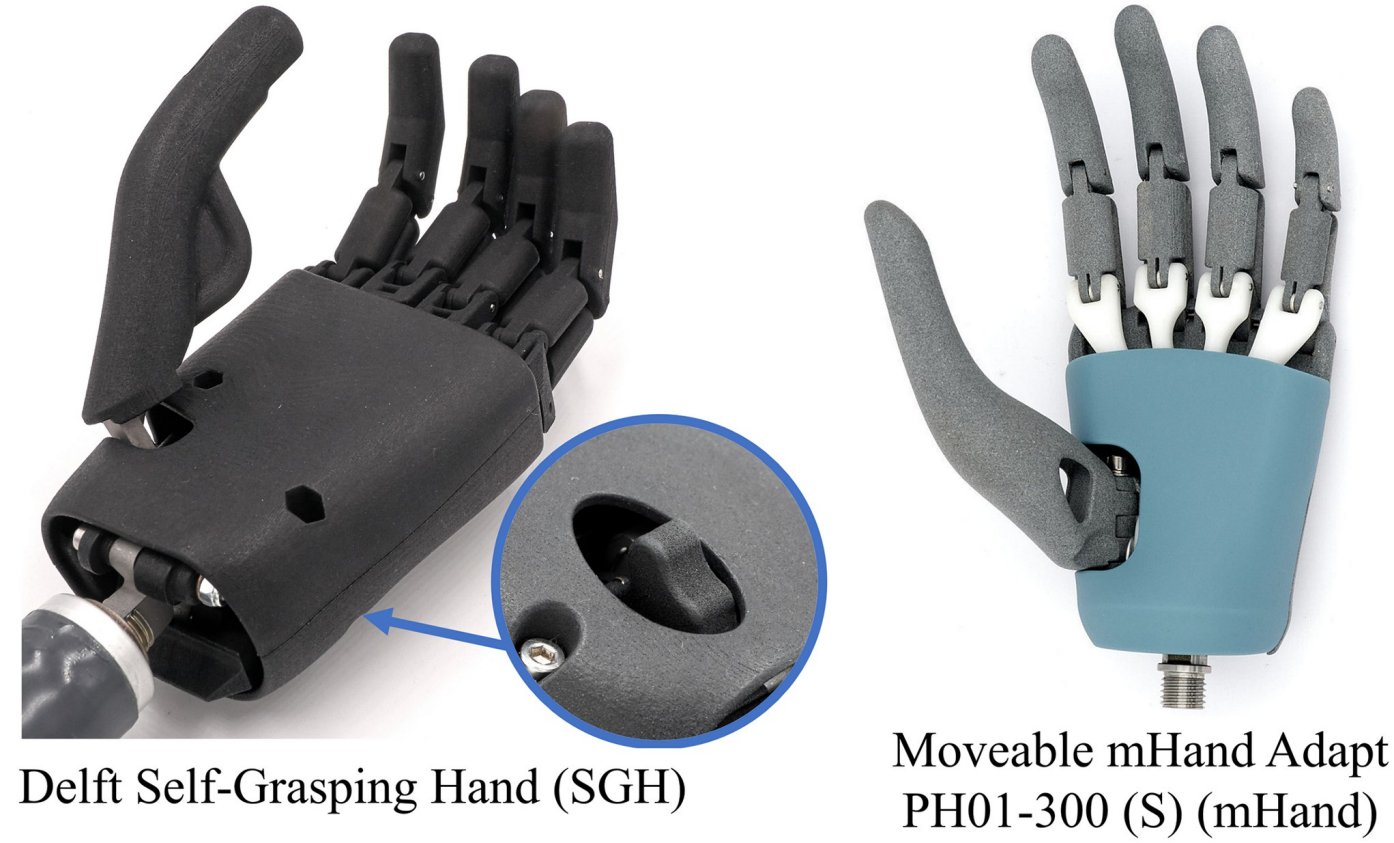
Images of SGH and mHand without any glove equipped. The circular window for the SGH highlights the button users must press to release the grasp. This button is located on the back of the hand. The mHand has no such button to release the grasp. The differences in finger shape are most visible around the tips of the fingers and thumb. Reproduced with permission from Gerwin Smit and Moveable [3].

The mHand is the commercialised version of a passive hand prosthesis prototype called the Delft Self-Grasping Hand (SGH) [4]. The two hands can be seen side by side in Fig 1. The SGH has a similar passive design to the mHand with three key differences. (1) The release mechanism is operated via a button on the back of the hand which requires the involvement of the contralateral hand during the release phase. This allows the SGH ratchet mechanism to be continuously engaged, and even further tightened, until the button is pressed unlocking the ratchet. This is in contrast to the mHand which switches between grasping and releasing each time the wrist goes from a resting state into a state of increased extension. The mHand therefore does not necessarily require the involvement of the contralateral hand. (2) The joints of the mHand’s fingers were more flexible than those of the SGH and overall the fingers of the mHand have a slimmer more rounded shape especially around the fingertips. This gives the fingers a more natural shape. (3) Both hands offer a similar range of motion for thumb adduction/abduction and friction based position holding mechanism. However the SGH additionally allows the thumb to rotate along the axis of the digit where as the mHand does not offer this motion.

Previous studies explored the performance of the SGH with both anatomically intact participants using a prosthesis simulator [4], and with prosthesis users making use of the hand in their day-to-day lives [5]. Both studies highlighted the release switch as a limitation to the design of the SGH. The mHand therefore employs an alternative releasing mechanism. This paper expands upon the work in Chadwell et al. [4] which explored the involvement of the contralateral hand in the operation of the SGH. When using passive prostheses, it is common for the contralateral hand to be involved in aiding the prosthesis with completing tasks [5]. If the contralateral hand is required to interact with the prosthesis, it will be unavailable for other tasks such as carrying an object. This extra input is important to monitor as less involvement of the contralateral hand may result in a better user experience. This paper aims to determine whether the new mHand prosthesis reduces users’ reliance on their contralateral hand. In addition, user performance is assessed via the Southampton Hand Assessment Procedure to determine if they are more or less competent with the mHand over the SGH.

## Method

In this study, comparisons were drawn to existing data from a study of the SGH [4]. The methodology for data capture was matched as closely as possible to that described in the previous paper. Functionality was assessed using the Southampton Hand Assessment Procedure (SHAP) [6]. 10 repeats of the SHAP were undertaken by each participant. Contralateral hand use during grasp and release was calculated based on video data recorded during the performance of the SHAP tasks. Further detail is provided below.

### Participants

Ten participants were invited to take part in the testing of the mHand. They were all right-handed, anatomically intact, and had no upper-limb impairments. As the previous trial had limited information on the demographics of its participants, it was decided to source individuals for the mHand trial from the same location as those who took part in the SGH trial, the University of Salford [4]. Though this does not guarantee the populations to be equivalent, it is believed that this would aid in achieving this goal. There was an even split of male and female participants and the age of the participants ranged from 20 to 35. This is a similar age range to those included in the SGH trial however the SGH trial included 3 male and 7 female participants [4]. University of Salford Health Research Ethics committee granted ethical approval for the trial (Ref. 9606) and informed consent was received from every participant. Participants were recruited between the 6^th^ of February 2023 to the 23^rd^ of February 2023. SGH data is available for ten right-handed, anatomically intact participants can be found in this linked paper (DOI: 10.1371/JOURNAL.PONE.0252870) [4].

### Prosthesis and simulator

The same right-handed, Moveable mHand Adapt PH01–300 (S) passive hand prosthesis was used by all participants for all tasks. This hand is a similar size to the SGH. As all participants had intact upper limbs, they wore the TRS body-powered prosthesis simulator (TRS Inc, Boulder, Colorado, USA) to which the mHand was attached allowing them to use the prosthesis to complete the tasks. The harness and cable system were removed from the simulator as these were not required. The mHand was covered with the Steeper F(T)SGL6 silicone glove (Steeper Group, Leeds, United Kingdom) throughout the trial. The simulator equipment and mounting mechanism were the same as was used during the SGH trial [4].

### Southampton Hand Assessment Procedure (SHAP)

The Southampton Hand Assessment Procedure (SHAP) encompasses 26 different tasks and the participant must complete each task as quickly as possible [7]. These exercises are completed by the participant whilst seated at a table. There are two types of tasks within the SHAP protocol: abstract tasks and Activities of Daily Living (ADL) tasks. The 12 abstract tasks involve moving objects including cylinders, flat plates, and spheres. The aim is to quantify the performance of a hand under six identified grip types; tip, lateral, tripod, spherical, power, and extension [6, 7]. The 14 ADL tasks allow for testing of the prosthesis across a range of common ‘daily living’ tasks such as pouring liquid out of containers, turning a key, undoing buttons and more. Times for each task are recorded and fed to the SHAP website to generate an Index of Functionality (IoF) score also referred to as a SHAP score [8]. Task time is limited to 100 seconds and any attempt taking longer than this is recorded as 100 seconds. This score is then normalised to 100 where 100 would equate to a normally functioning hand completing the tasks. This SHAP score provides a quantitative way to measure the performance of a prosthesis and to compare different devices [6]. Participants were asked not to use their contralateral hand unless they felt it was necessary. This was all in keeping with the method of the SGH trial.

#### Alterations made to SHAP protocol

In the previous trial with the SGH, alterations were made in order to make the SHAP procedure feasible to complete. This included removing seven tasks from the protocol including the abstract task of moving the heavy spherical object and the ADLs of picking up coins, button board, page-turning, jar lid opening, and the lifting of the light and heavy object. This was because the SGH could not complete these tasks. During pilot testing with the mHand, it was found that some of these tasks were more feasible to complete with the mHand. These were the ADLs of picking up coins, button board, page-turning and jar lid opening and hence these tasks were reincluded into the test suite for mHand testing. The remaining three tasks, moving the heavy spherical object and lifting the heavy and light object, were still not feasible and were thus excluded from testing. For the heavy sphere, this was because the mHand was not able to get a good enough grip on it. For lifting the heavy and light objects, this was due to the aperture of the mHand not being large enough to wrap around the jar. In addition, following reflections from the SGH trial, the simulated food-cutting task was excluded due to the difficulty of accomplishing the task. A wide range of techniques had been used to accomplish the task leading to uneven, highly variable results and damage to the prosthesis [4]. Methods sometimes involved holding the blade end into the tip of the thumb or palm and over time these areas of the glove developed damage. As the mHand has a similar glove over a hard plastic exterior, it was decided that to protect the performance of all participants, this task would be excluded. Table 1 summarises the SHAP tasks which were included and excluded. Consistent with the approach used in the SGH study [4], where a task was excluded, the maximum time of 100 seconds was input into the online SHAP system for SHAP score generation.

**Table 1:** SHAP tasks there were included and excluded from the testing protocol.

Abstract Object Tasks				Activities of Daily Living (ADLs)			
1. Light spherical	✓	7. Heavy spherical	✗	13. Pick up coins	✓	20. Lifting a heavy object	✗
2. Light tripod	✓	8. Heavy tripod	✓	14. Button board	✓	21. Lifting a light object	✗
3. Light power	✓	9. Heavy power	✓	15. Simulated food-cutting	✗	22. Lifting a tray	✓
4. Light lateral	✓	10. Heavy lateral	✓	16. Page turning	✓	23. Rotate a key	✓
5. Light tip	✓	11. Heavy tip	✓	17. Jar lid	✓	24. Open/close zip	✓
6. Light extension	✓	12. Heavy extension	✓	18. Glass jug pouring	✓	25. Rotate a screw	✓
				19. Carton pouring	✓	26. Door handle	✓

Numbering indicates the order the tasks were completed in. ✓ indicates inclusion into this trial and a ✗ indicates exclusion from the trial.

### Contralateral hand involvement

Along with the SHAP score, a measure for the amount of contralateral hand involvement was also collected. Contralateral hand involvement was split into 4 categories; direct and indirect interactions during grasp, and direct and indirect interactions during release. Direct interactions refer to the participant touching the prosthesis itself whereas indirect interactions apply to when the participant interacts with the object being manipulated to place it into or remove it from the prosthesis. Definitions for each interaction type are provided below. These are taken from the previous paper [4] and slightly updated to account for the change in device and its altered mechanism. Additions are highlighted in bold.

- Direct interaction during grasp: contralateral hand contacts the prosthesis to adjust **or activate** the grasp. **This also includes reopening the hand if the participant immediately readjusts or engages the grip on the object**.
- Indirect interaction during grasp: contralateral hand stabilises an object so that the prosthetic hand can push the object into itself, or the contralateral hand pushes the object into the prosthetic hand. **This also includes reopening the hand if the participant immediately readjusts or engages the grip on the object**.
- Direct interaction during release: contralateral hand contacts the prosthesis to activate the releasing mechanism **and terminate the grasp.**
- Indirect interaction during release: contralateral hand removes an object from the prosthesis, or uses an object or surface to activate the releasing mechanism **and terminate the grasp.**

The length of these interactions was calculated from the first motion of the contralateral hand toward the prosthesis or object to perform one of the four interactions, until the moment the contralateral hand stopped that interaction.

Consistent with our previous study [4], some interactions of the contralateral hand were not recorded as they were considered necessary supporting actions. These included stabilising the button board backplate, glass jar, arrow bracket, and SHAP case. Additionally, assistive actions were also not counted such as aiming the screwdriver tip into the screw head, holding the other side of the tray during the transportation phase (i.e. outside of helping to grasp/release it), shifting the case/board to a more ergonomic position, and resetting tasks to their original state if it was not possible to continue the task from its current state (i.e. tripod/tip/extensions task objects fell over and when the coins fell off the SHAP tray). If attempts took longer than 100 seconds those data points were excluded from the contralateral hand interaction data set. These methods are consistent with those from the SGH trial.

To enable the analysis described above, a video of the participant completing the tasks was recorded. Two cameras were used pointing toward the task completion area from two different perspectives. This reduced the possibility of the user occluding the view of the cameras, a phenomenon that was noted during the SGH trial [4]. The video footage was later reviewed manually to analyse how long each interaction took, utilising a specially written MATLAB script which automated part of the process. The accuracy of the times collected is estimated to be within ± 3 frames equating to ±100 milliseconds, as videos were shot at 30 frames per second. Note that it was possible for no contralateral interaction to take place, as the mHand can be opened and closed without the need for the contralateral hand. For example, during the button board tasks, participants could push the thumb of the mHand against the table to close the hand and repeat this action to open it thus not requiring contralateral hand interaction. It was also possible for both direct and indirect actions to occur at the same time. An example of this sometimes occurred when manipulating the glass jug. Some participants spanned their contralateral hand across both the jug and thumb of the mHand and would then close their contralateral hand thus tightening the grip of the prosthesis around the handle of the jug. In this instance, the interaction would be recorded simultaneously as a direct and indirect grasp during the period between the contralateral hand reaching for the jug and finally releasing the jug.

### Testing protocol

Each participant undertook the trial in a quiet, distraction-free space with a table large enough to accommodate the SHAP tasks and a chair for them to sit on. The participant was shown how the opening and closing mechanism of the mHand works, after which they had the opportunity to put on the simulator and make any adjustments to make it more comfortable. After this, the participant removed the simulator and was shown how to do tasks 1 to 6 in order, using the mHand, by the experiment attendant. They were shown how to do each task only once and were instructed they could complete the task with any method using the mHand they liked as long as they refrained from using their contralateral hand if possible. The participant could then don the simulator again and had two practice runs of these first six tasks. Verbal assistance was given if the participant was struggling with a task. After this, the participant redid the tasks under test conditions with this attempt being recorded. These steps of showing the participant each task once, allowing them to practice twice, and then recording the attempt apply for the remaining tasks, separated into three further task blocks (8–12, 13–19, and 22–26). Once the participant had completed one full run-through of the SHAP tasks they were then asked to redo the whole set of SHAP tasks, in order, another nine times. During this time they were not shown how to do the tasks again nor was other assistance towards completing the tasks provided. Participants were allowed to have breaks after each attempt to adjust the simulator or otherwise rest their arm. They were allowed to pause between tasks if they felt they needed to adjust something immediately.

### Statistical analysis

All statistical analysis methodologies were conducted in as similar a manner as possible to those performed in the SGH trial. This allows for a direct comparison between the two data sets to take place. As explained previously, there are two areas of interest being investigated; SHAP scores and contralateral hand involvement. These areas are further divided into the analysis of the mHand itself and the comparison between the mHand and the SGH. The statistical analysis of each of the four permutations is explained below.

#### SHAP scores of the mHand itself

SHAP scores for each attempt were calculated using the SHAP website [8]. Two paired samples t-tests were conducted on the data. The first was between the first and last three attempts and was to determine if there had been an improvement in the SHAP score. We hypothesised that the SHAP score would be higher for the last three attempts than the first three. The second was to determine if a clear plateau in performance could be seen in the data set. This involved conducting paired samples t-tests between the 10^th^ attempt and each previous attempt individually (first the 9^th^ attempt then 8^th^ and so on) until statistical significance was identified. Once this is reached the last attempt that had no statistically significant difference is taken as the start of the plateau. The previous SGH trial saw a plateau after the 5^th^-6^th^ attempt.

#### SHAP scores comparison between the mHand and the SGH

When comparing the mHand and SGH data sets, unpaired samples t-tests were used to compare overall mean SHAP scores for each hand. In addition, the means of the first and last three attempts were also compared using unpaired samples t-tests. It was hypothesised that the mHand would achieve higher SHAP scores than the SGH device.

#### Contralateral hand involvement with the mHand itself

As analysing the relevant contralateral hand interaction data was time-consuming, only videos for the first and last three attempts were analysed. For all comparisons conducted in the Results section, a paired samples t-test, Cohen’s d effect size, and z-values for Skewness and Kurtosis were computed. Additionally, a grand mean was calculated. A grand mean is derived by determining the mean for each participant, over every included attempt, then finding the mean over all the participants. A grand median is also computed utilising a similar methodology. All these comparisons and methods are equivalent to what was done during the SGH testing. It was hypothesised that contralateral hand involvement would reduce between the first three and last three attempts.

#### Contralateral hand involvement comparison between the mHand and the SGH

Comparisons between the mHand and the SGH were performed using independent samples t-tests. This was done as the two data sets do not share the same population. Additionally, grand means and medians were also calculated using the same method as described above. It is hypothesised that the mHand will require less contralateral hand involvement than the SGH. Information on Cohen’s d effect size and z-values for Skewness and Kurtosis were also determined.

#### Statistical significance

P-values from the t-test were considered significant if *p* < 0.05. Z-values for skewness and kurtosis were significant if |*z*| > 1.96 (*p* < 0.05) or |*z*| > 2.58 (*p* < 0.01) [9]. Outliers would be labelled according to the outlier labelling rule 2.2*IQR [10]. If normality assumptions were infringed upon this was highlighted. T-testing was still conducted as they are tolerant to such violations with respect to Type I error [11–14]. When using an unpaired samples t-test, it was assumed that variance was roughly equivalent between the trials.

## Results

The results of this trial are split into two sections both of which analyse comparisons between the mHand and the SGH devices. One section examines SHAP score results and another looks at comparisons for contralateral hand interaction during SHAP tasks. Additionally, there is a section highlighting qualitative elements observed during testing such as lack of damage to the mHand, the difficulties encountered when completing some tasks, grips used by participants, and more. As the testing for the SGH and the mHand involved different tasks, the full data sets representing the respective prostheses are not directly comparable. Because this paper focuses on the comparison between the two prostheses, results for the full mHand data set are provided in S1 Appendix.

### Comparison between mHand and SGH SHAP scores

In total, 100 SHAP scores were analysed (10 participants each with 10 attempts). The precision of timekeeping for the SHAP scores was to the hundredths of a second. Regardless of the data set, the same number of data points are present however the score values change if you are looking at the full data set, for either hand, or the overlapping one. This is because the full and overlapping data sets have different numbers of included tasks which will affect the overall scores. Hence, only overlapping task sets are analysed in this comparison however a discussion of the full mHand SHAP scores can be seen in S1 Appendix.

**Fig 2:**
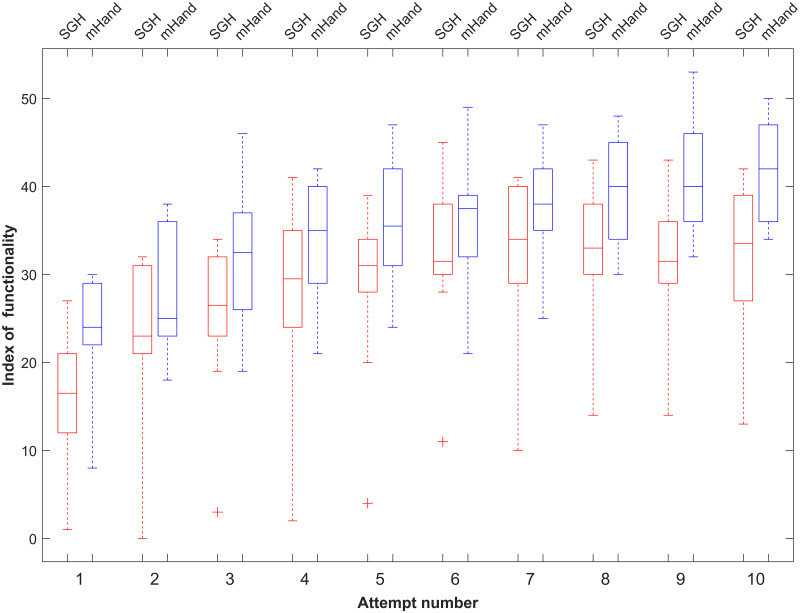
mHand and SGH SHAP scores over successive attempts. Data of participants from the mHand and SGH were graphed together within this box plot. This figure analyses the overlapping task data from the mHand and SGH data sets. For each attempt, the line within the box represents the median, the upper and lower edges of the box represent the upper and lower quartiles respectively, and the ends of the whiskers are the maximum and minimum values.

The mHand and SGH participants began at roughly the same SHAP score level, evidenced by no significant difference between the two data sets over the first three attempts (*t*(9) = −1.875, *p* = 0.077, *d* = −0.84) (Fig 2). There was a learning period visible for both devices over the ten attempts however the mHand had a greater upward trajectory than the SGH. The difference in SHAP scores for the last three attempts shows significance (*t*(9) = −2.887, *p* = 0.010, *d* = −1.291) with the mHand achieving higher grand mean scores of 40.83 ± 5.63s compared to 32 ± 7.87s for the SGH (Table 2). mHand users could achieve maximum scores of 53 versus the highest SGH score of 45 which also supports the conclusion that mHand is a more functional prosthesis. Furthermore, the standard deviation was reduced considerably during the mHand trials as compared to SGH and there were no outliers in mHand data.

**Table 2:** Statistical compassion between SGH and mHand SHAP scores.

	Hand Prosthesis	Grand Mean (±SD) [s]	Paired Samples t-test	Cohen’s d (d =)	Shapiro-Wilks test of normality	Skewness and Kurtosis (z values)
All SHAP scores between SGH and mHand	SGH	27.99 ± 8.59	t(9) = -2.037	-0.91	W(10) = 0.962	Skew: -0.72
	mHand	34.81 ± 6.19	p = 0.057		p = 0.825	Kurt: -0.27
First 3 attempts between SGH and mHand	SGH	21.00 ± 8.53	t(9) = -1.875	-0.84	W(10) = 0.930	Skew: -0.31
	mHand	27.43 ± 6.71	p = 0.077		p = 0.447	Kurt: -0.14
Last 3 attempts between SGH and mHand	SGH	32.00 ± 7.87	t(9) = -2.887**	-1.29	W(10) = 0.911	Skew: -1.15
	mHand	40.83 ± 5.63	p = 0.010		p = 0.285	Kurt: -0.09

**Correlation significant to the 0.01 level.

*Correlation significant to the 0.05 level (no comparisons met this level).

For Kurtosis and Skewness: |*z*|>1.96 is significant when *p* < 0.05, and |*z*|>2.58 is significant when *p* < 0.01 [9].

### Comparison between mHand and SGH contralateral hand involvement

For mHand testing, 10 participants, 6 attempts (first and last three), and 22 tasks resulted in 1320 total different task attempts. However, there were nine excluded task attempts because those attempts ran over the 100 seconds task time completion limit bringing the total number of task data points to 1311. SGH testing saw the same number of participants and attempts (10 and 6 respectively) but only 19 tasks were included. Out of the 1140 data points, 32 were removed leaving 1108 total data points. However, when comparing the data of the two hands only the overlapping task data can be included. This means comparable data sets contain 18word different tasks leading to 1073 and 1060 analysed data points for the mHand and SGH respectively. All time data acquired during testing with the mHand can be found in the Excel spreadsheet provided (S1 File). This includes the time each participant took to complete each task and the amount of time the contralateral hand was involved in a task, including the type of involvement. Additionally, information such as which tasks were excluded (either for everyone or for an individual’s attempt) and the various iterations of SHAP scores are also provided.

#### Overall

When looking at total contralateral involvement, whilst the mean time of contralateral hand involvement for the mHand (4.70 ± 1.20s) is reduced when compared to the SGH (5.01 ± 1.82s), the t-test indicates this is not a significant difference (*t*(9) = 0.455, *p* = 0.654, *d* = 0.204) (Table 3). This indicates both devices are roughly similar with respect to contralateral hand involvement generally with a slight edge in favour of the mHand.

**Table 3:** Statistical comparison of contralateral hand interactions between mHand and SGH data.

	Hand Prosthesis	Grand Mean (± SD) [s]	Grand Median (± IQR) [s]	Paired Samples t-test	Cohen’s d (d =)	Shapiro-Wilks test of normality	Skewness and Kurtosis (z values)
Overall contralateral hand usage	SGH	5.01 ± 1.82	1.99 ± 1.82	t(9) = 0.455	0.204	W(10) = 0.986	Skew: -0.17
	mHand	4.70 ± 1.20	3.15 ± 1.13	p = 0.654		p = 0.990	Kurt: -0.06
Grasping interactions	SGH	2.73 ± 1.42	0.00 ± 1.70	t(9) = -1.882	-0.842	W(10) = 0.965	Skew: -0.10
	mHand	3.78 ± 1.05	1.98 ± 0.77	p = 0.076		p = 0.841	Kurt: -0.31
First 3 grasp interactions	SGH	3.28 ± 1.74	0.00 ± 2.37	t(9) = -1.860	-0.832	W(10) = 0.981	Skew: 0.02
	mHand	4.74 ± 1.78	2.28 ± 0.97	p = 0.079		p = 0.972	Kurt: 0.17
Last 3 grasp interactions	SGH	2.20 ± 1.22	0.00 ± 1.33	t(9) = -1.345	-0.601	W(10) = 0.911	Skew: 0.80
	mHand	2.83 ± 0.83	1.62 ± 0.63	p = 0.195		p = 0.286	Skew: -0.71
Release interactions	SGH	2.28 ± 0.46	1.50 ± 0.65	t(9) = 8.369**	3.743	W(10) = 0.960	Skew: -0.83
	mHand	0.91 ± 0.24	0.00 ± 0.73	p = <.001		p = 0.789	Kurt: 0.59
First 3 release interactions	SGH	2.52 ± 0.66	1.40 ± 0.77	t(9) = 6.761**	3.024	W(10) = 0.962	Skew: -0.64
	mHand	0.99 ± 0.28	0.00 ± 1.20	p = <.001		p = 0.811	Kurt: 0.40
Last 3 release interactions	SGH	2.05 ± 0.45	1.46 ± 0.65	t(9) = 7.412**	3.315	W(10) = 0.950	Skew: -0.12
	mHand	0.84 ± 0.24	0.00 ± 0.80	p = <.001		p = 0.673	Kurt: -0.51
Direct interactions	SGH	2.50 ± 1.14	1.30 ± 1.22	t(9) = -0.719	-0.322	W(10) = 0.949	Skew: 0.08
	mHand	2.86 ± 1.09	0.59 ± 2.27	p = 0.481		p = 0.662	Kurt: -1.08
Indirect interactions	SGH	2.51 ± 1.32	0.00 ± 0.00	t(9) = 1.521	0.680	W(10) = 0.886	Skew: 1.74
	mHand	1.84 ± 0.45	0.00 ± 0.00	p = 0.146		p = 0.154	Kurt: 1.13

**Correlation significant to the 0.01 level.

Correlation significant to the 0.05 level.

For Kurtosis and Skewness: |*z*|>1.96 is significant when *p* < 0.05, and |*z*|>2.58 is significant when *p* < 0.01 [9].

#### Grasp vs release

**Fig 3:**
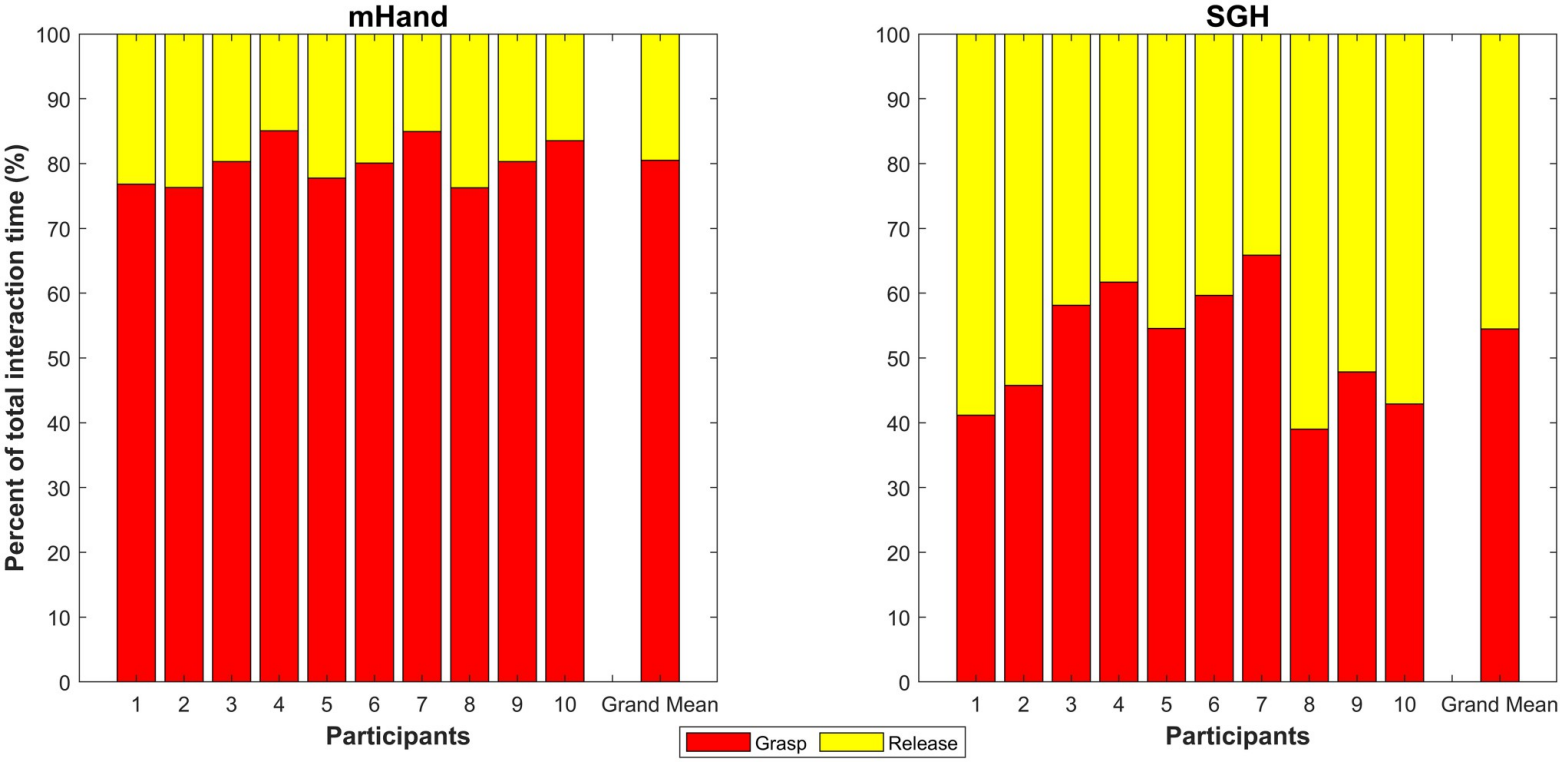
Contralateral hand involvement comparison during grasp and release interactions between mHand and SGH. Each column encompasses the total interaction time, for each participant, over every analysed attempt. This is normalised to 100%. The rightmost column shows the grand mean of the entire data set. Grasp data (red) is graphed below release data (yellow).

SGH data showed a more or less even split in time between grasping and releasing interactions (Fig 3). The mHand had a higher proportion of time spent on grasping interactions and a lower time spent on releasing interactions when compared to the SGH. This is true for the overall data set and for the first and last three attempt data sets. However, out of these mean comparisons, only the release data sets are significantly different with all of them achieving P-values of less than 0.001. Additionally, it is notable that, when looking at the grand medians the SGH had values of zero for all grasp comparisons. This indicates over half the SGH participants, over half the time, could grasp objects without needing any contralateral hand involvement. This was not the case with the mHand. However, all mHand release comparisons did have a grand median of zero indicating that at least half the participants were able to release the objects over half the time without using their contralateral hand. This is in contrast to the SGH which had non-zero grand medians for all release comparisons. These trends lend credence to the idea that mHand users have to interact with the prosthesis more during the grasp phase of tests but less during the release phase when compared to the SGH.

#### Direct vs indirect

Here trends are less pronounced. Generally, there is more time spent on direct interactions with the mHand than the SGH and slightly lower indirect interactions for the mHand. This trend, however, is not significantly different indicating little difference between the devices on this matter. Grand medians for both hands indicate that over half the participants did not conduct any kind of indirect contralateral hand interaction with the prosthesis.

### Additional observations

Unlike in the SGH trial, the glove of the mHand stayed intact throughout the duration of testing. Additionally, the hand itself experienced no damage or other noticeable reduction in function throughout testing. Though the mHand’s small aperture limited which SHAP tasks it could do, just like the SGH, the changes to a slimmer, more pointed shape of the thumb and digits meant tasks such as picking up the coins and page-turning were possible to do in a natural way. Another note is that the ratchet mechanism would sometimes release unexpectedly during the glass jug pouring task and the screwdriver task. The cause of this is unknown but it is potentially due to overloading of the ratchet mechanism. This behaviour was not noted during the SGH trial. Similar to observations during the SGH trial, for many tasks, participants had to awkwardly shift their body or right arm in order to accomplish the tasks. This included raising their right elbow extremely high or leaning the torso significantly. This discomfort seemed to push many participants to stand or otherwise raise their bodies slightly. Unfortunately, the simulator used did not have a working wrist rotator function which may have helped with this. The lack of a wrist rotator was present in both trials hence the data is still comparable.

When analysing the grip used (S2 Table in S1 Appendix), it was noted that there was little difference between a tip, tripod, and extension grip as the prosthesis only moved the fingers closer to the palm and did not manipulate finger shape or position individually. This was especially evident when comparing the grip between the tip and extension tasks. The gripping motion was generally the same however, for the extension task, the object reached further into the hand aperture. Minimal, if any, support was provided by the palm or sides of the fingers. Because of this, these types of grips will be referred to collectively as pinch grips. Grasps used would sometimes differ from the intended grip identified by SHAP. This was especially prevalent for tasks intended for lateral grips as participants usually preferred pinch grips. This contrasts with the SGH test where participants more often used lateral grips, even using it for tasks for which this grip was not the intended one.

Amongst the most difficult tasks were pickup coins, glass jug pouring, rotating a key, and opening/closing the zip. These tasks required either a high level of grip positioning or a precise technique or both to complete. Due to this, participants often struggled to find the ideal grip, especially during initial attempts.

## Discussion

### SHAP scores

The higher scores over the last three attempts achieved by the mHand indicate that it is a more functional hand compared to the SGH. This matches what we hypothesised for mHand performance. However, the lack of a clear performance plateau indicates participants may take longer to learn effective hand usage than the SGH. This, though, does not seem to be a major issue as the SHAP scores over the first three attempts for the mHand were slightly higher than those of the SGH indicating the hand is at least just as capable from the outset of usage as the SGH if not slightly more so.

### Contralateral hand involvement

When looking at the mHand data, it is clear there is proportionally more contralateral involvement in grasping than in releasing. This is also clearly different from the results of the SGH trial which showed little clear difference between grasp and release interactions. The principal reason why more time is spent on grasp interactions may be due to participants needing to readjust their grasp. As the mHand is not able to further tighten a grip once a position is set, if a participant needs to achieve a firmer grip, they need to completely open the hand and reclose it. This opening and retightening would sometimes lead to the participant using the same ratchet setting numerous times and thus needing to repeat the process until they realised they had to go one more step. This ultimately had some participants struggling to get an initial good grip. This is one area the mHand is inferior to the SGH as it caused participants to become frustrated and led to some inconsistencies if the participants were not as attentive to how many clicks were heard from the ratchet. This additional cue may also indicate why a performance plateau was not clearly reached as participants needed longer to get a feel of a good grasp with the mHand. One solution is to ensure this flaw is highlighted to users, as once people knew how to use audio cues to identify the tightness of the grip they were able to immediately readjust rather than continue with their attempt only to realise the grip was not solid enough. This design strategy is ultimately a trade-off as, on the one hand, it requires more precision when selecting a grip strength during the grasp but, on the other hand, it does allow the participants to release an object without having to touch the mHand. This second point leads to why release interactions for the mHand are much lower than those of the SGH. It was very easy for participants to open the mHand without touching it by pressing against the table or object itself. This, combined with a request to use the contralateral hand as little as possible, meant participants were encouraged to use their contralateral hand for a limited number of tasks. This is in comparison to the SGH where participants had to touch the prosthesis to release it for every task. Additionally, as mHand participants only needed to touch the hand during a few tasks, a performance floor seemed to be reached quickly as overall there is little difference in release interaction times between the first and last three attempts. This means that participants are likely able to achieve peak release performance more quickly, offsetting the increased learning curve for initially grasping objects.

Direct vs indirect comparisons lead to fewer concrete conclusions, however, there did seem to be more direct and less indirect interactions with the mHand than the SGH. One reason for this may be that users were more comfortable manipulating the mHand by directly touching it. This potentially stems from the fact that it is more difficult to achieve an initial grasp with the mHand and thus participants felt they had a better understanding of the tightness of the grip if they touched the hand directly. Alternatively, this may come from how the participants were shown to use the hand as the demonstrator preferred to use direct interactions when manipulating the hand thus swaying users to do the same. In any case, direct and indirect interaction comparisons between the mHand and SGH did not show any significant differences hence it is difficult to draw concrete conclusions when comparing the hands.

### Additional observations

When examining grip types used, the main observation was the lack of lateral grips used during the tasks. This potentially comes from two areas. One is the design of the thumb locking mechanism being purely friction-based and thus was not able to hold a tight grip. This is contrasted to pinch grips which could generate much stronger grips. The other factor may be due to participants being instructed to refrain from using their contralateral hand where possible. As it was not possible to use a lateral grip without touching the prosthesis, whereas pinch grips are possible, participants opted to use pinch grips. Additionally, participants mostly used one of the six SHAP intended grips (tip, lateral, tripod, spherical, power, and extension) which indicates the participants potentially did not feel limited by the hand’s intended grasping style. It is also important to note the awkward angles they would have to hold their arm in order to manoeuvre the prosthesis into the correct position for some tasks may also impact how well they were able to complete the task. It is possible the addition of a wrist rotator for the prosthesis might improve results further but as that would not make the two trials comparable future work can be undertaken to analyse this factor.

## Limitations of the trial

The general limitations of this trial include the limited population pool of 10 participants. More participants would be able to provide clearer data trends as well as reduce the influence of outlier results. Additionally, these participants were not proficient hand prosthesis users. The focus of this trial was to compare the performance and interactions between the two hand prostheses however investigating how proficient users would use the mHand would be an interesting avenue for future work.

One limitation of the comparisons is the restrictive, 10 participant, population pool involved in both this trial and the previous SGH trial. A greater number of participants would provide clearer data trends as well as reduce the influence of outlier results and biases of individual participants. It was, unfortunately, not possible to include more participants within the available study time period. Another note is the dissimilarity between the cohorts of the two trials. The mHand trial involved 5 females and 5 males whilst the other had 7 females and 3 males. This, however, did not seem to influence results as both sexes had similar performance characteristics. Other demographic differences were not recorded and thus were not possible to account for. Despite this, it is believed the mitigation strategy of having a similar age range and involving only students from the University of Salford for both trials helps to alleviate this point somewhat. Additionally, by limiting the participant pool to anatomically intact individuals, this also makes the results difficult to link to the wider population of prosthesis users. Future work is required to understand how prosthesis users would use the device in general.

Another caveat relates to the differences in included tasks between the two trials. The mHand data set included 22 tasks whilst the SGH trial included 19. This 3-task difference meant participants in the mHand trial had more time using the prosthesis over those testing with the SGH. This may result in comparisons being skewed in favour of the mHand in the form of improved SHAP scores. However, it is also possible that the increased time may also lead to greater fatigue and thus lower SHAP scores for mHand users. How these two effects interact with each other is unclear but it is expected that due to their contradictory effects, bias one way or another would have limited effect on the remaining results.

During the trial, some issues arose that required some slight alterations to test equipment. After participant 3 completed testing, the carton used for task 19 began to leak. In response, a new carton of equivalent size and material was purchased to replace it. As the original carton was well used the new carton was deformed in order to mimic the original’s dimensions. During participant 5’s testing, a hole opened up next to the smallest buttonhole causing confusion and difficulty finishing the task for the first 2 attempts. After some stretching the hole became as big as the original button hole and thus, during their button board task, it was judged that if the button went into either hole it was considered valid for task completion. After participant 5, the new hole was sewn closed for the remaining participants thus successive attempts would not have been affected. Finally, during task 25 a misunderstanding caused participant 1 to release the screwdriver in the wrong location. This will have caused slightly reduced attempt times for this task and participant. This task was conducted correctly for all other participants. For all these small discrepancies, it was deemed that though they may have affected results slightly, their impact would have been negligible and thus these data points were kept as valid.

## Conclusion

This is the first study that conducts an in-depth comparison of quantitative hand prosthesis functionality and contralateral hand involvement between two different adjustable hand prostheses. The mHand hand prosthesis is a new, commercialised version of the SGH. The mHand had a higher overall grasp interaction time of 3.78 ± 1.05s compared to that of the SGH with 2.73 ± 1.42s. This difference was not statistically significant. However, there was a statistically significant reduction in release interactions in favour of the mHand from over two seconds to under one second. Overall, this results in a near 40% drop in contralateral hand interaction times from the SGH to mHand. Additionally, the mHand saw a 27.6% higher SHAP scores over the last three SHAP attempts when compared to the SGH. The improved SHAP scores and reduced contralateral interaction times likely come from the altered locking mechanism, hand geometry, and added flexibility. Though slightly more difficult to use when grasping an object, the mHand allowed users to quickly release objects with over half of users not requiring the contralateral hand to do so. Overall, data indicates the mHand is a hand prosthesis which offers improved functionality and reduced contralateral hand interaction time compared to its previous version.

## Supporting information

S1 Appendix Results of full mHand data set. (PDF)

S1 Fig All mHand SHAP scores over successive attempts. Data from all participants were combined into this box plot to show scores for each successive attempt. This figure analyses the complete mHand data set. For each attempt, the line within the box represents the median, the upper and lower edges of the box represent the upper and lower quartiles respectively, and the ends of the whiskers are the maximum and minimum values.
(TIF)

S2 Fig mHand contralateral hand involvement during grasp and release interactions. Each graph represents one participant’s data for grasp and release interactions over the first or last three SHAP attempts. ‘G’ labels represent grasp interactions, ‘R’ labels represent release interactions. The numbers following ‘G’ or ‘R’ represent the series of attempts analysed. Whiskers represent the standard error.
(TIF)

S3 Fig mHand contralateral hand involvement during direct and indirect interactions. Each graph represents one participant’s data for direct and indirect interactions over the first or last three SHAP attempts. ‘D’ labels represent direct interactions and ‘I’ labels represent indirect interactions. The numbers following ‘D’ or ‘I’ represent the series of attempts analysed. Whiskers represent the standard error.
(TIF)

S1 File Raw data. Spreadsheet of contralateral hand interaction data for the mHand and SHAP scores for the mHand and SGH.
(XLSX)
