# Correction: A quantitative comparison between the mHand Adapt passive adjustable hand prosthesis and its predecessor, the Delft Self-Grasping Hand

**DOI:** 10.1371/journal.pone.0322238

**Published:** 2025-04-04

**Authors:** Spyros L. L. Krinis, Alix Chadwell, Laurence Kenney, Gerwin Smit

In the Comparison between mHand and SGH contralateral hand involvement subsection of Results, there is an error in the sixth sentence of the first paragraph. The correct sentence is: This means comparable data sets contain 18 different tasks leading to 1073 and 1060 analysed data points for the mHand and SGH respectively.

In the conclusion section there is an error in the fifth sentence. The correct sentence is: Overall, this results in a 60% drop in release contralateral hand interaction times from the SGH to mHand.

S1 Fig is uploaded incorrectly. Please view the correct S1 Fig here.

## Supporting Information

S1 Fig All mHand SHAP scores over successive attempts.
Data from all participants were combined into this box plot to show scores for each successive attempt. This figure analyses the complete mHand data set. For each attempt, the line within the box represents the median, the upper and lower edges of the box represent the upper and lower quartiles respectively, and the ends of the whiskers are the maximum and minimum values.
(TIF)
