# Supplementary material for: A quantitative comparison between the mHand Adapt passive adjustable hand prosthesis and its predecessor, the Delft Self-Grasping Hand
Source: PLoS One. 2024 Mar 21;19(3):e0300469. doi: 10.1371/journal.pone.0300469 (PMC10956796; doi:10.1371/journal.pone.0300469)
Supplement: S1 Appendix — (PDF) [file pone.0300469.s001.pdf]

Supporting information

S1 Appendix. Results of full mHand data set.

mHand Only SHAP Scores

Participants' SHAP scores increased significantly over the ten attempts at SHAP using the mHand (first three attempts: grand mean = 31.1 ± 4.01; last three attempts: grand mean = 48.07 ± 1.36; ($t(9) = 10.032,\ p < 0.001,\ d = 3.172$) (S1 Fig). This learning curve seemed to last for a majority of the time however, when looking for a plateau in performance, the plateau seemed to begin at the 8th attempt. The more limited plateau period, when compared to the previous trial's period between attempts 6 and 10 [4], leaves open the possibility that a plateau in performance was not achieved and that more learning may be achievable.

S1 Fig. All mHand SHAP scores over successive attempts. Data from all participants were combined into this box plot to show scores for each successive attempt. This figure analyses the complete mHand data set. For each attempt, the line within the box represents the median, the upper and lower edges of the box represent the upper and lower quartiles respectively, and the ends of the whiskers are the maximum and minimum values.

mHand Only Contralateral Hand Involvement

Overall

There was a significant reduction in total contralateral interaction time between the first and last three attempts going from 5.62 ± 2.23s to 3.6 ± 1.25s ($t(9) = 3.683,\ p = 0.005,\ d = 1.165$) seen in S1 Table. This follows data seen during the SHAP scores section indicating participants became more proficient at using the device over time.

S1 Table. Complete contralateral interactions: mHand statistical data. (mHand only data)

	Data sets	Grand Mean (± SD) [s]	Grand Median (± IQR) [s]	Paired Samples t-test	Cohen's d (d =)	Shapiro-Wilks test of normality	Skewness and Kurtosis (z values)
Overall contralateral hand usage	First 3	5.62 ± 2.23	2.75 ± 1.12	t(9) = 3.683**	1.165	W(10) = 0.847	Skew: 2.12
	Last 3	3.60 ± 1.25	1.78 ± 1.52	p = 0.005		p = 0.054	Kurt: 1.29
Grasping vs Releasing interactions	Grasping	3.48 ± 1.32	1.52 ± 1.17	t(9) = 6.940**	2.195	W(10) = 0.944	Skew: 1.19
	Releasing	1.12 ± 0.30	0.00 ± 0.77	p = <.001		p = 0.602	Kurt: 0.96
Grasping interactions	First 3	4.40 ± 1.95	1.85 ± 1.05	t(9) = 3.572**	1.129	W(10) = 0.867	Skew: 1.84
	Last 3	2.58 ± 0.99	0.92 ± 1.35	p = 0.006		p = 0.093	Kurt: 0.80
Releasing interactions	First 3	1.23 ± 0.34	0.45 ± 1.2	t(9) = 2.892*	0.914	W(10) = 0.884	Skew: 0.68
	Last 3	1.02 ± 0.29	0.00 ± 0.88	p = 0.018		p = 0.146	Kurt: 1.32
Direct vs Indirect interactions	Direct	3.07 ± 1.48	1.18 ± 2.3	t(9) = 3.362**	1.063	W(10) = 0.941	Skew: 0.85
	Indirect	1.54 ± 0.34	0.00 ± 0.00	p = 0.008		p = 0.565	Kurt: -0.42
Direct interactions	First 3	3.87 ± 2.00	2.15 ± 2.63	t(9) = 3.648**	1.154	W(10) = 0.853	Skew: 2.06
	Last 3	2.27 ± 1.15	0.47 ± 2.3	p = 0.005		p = 0.063	Kurt: 1.17
Indirect interactions	First 3	1.75 ± 0.52	0.00 ± 0.00	t(9) = 2.544*	0.805	W(10) = 0.926	Skew: -1.17
	Last 3	1.33 ± 0.31	0.00 ± 0.00	p = 0.031		p = 0.411	Kurt: 0.93

**Correlation significant to the 0.01 level.
*Correlation significant to the 0.05 level.
For Kurtosis and Skewness: $|z| > 1.96$ is significant when $p < 0.05$, and $|z| > 2.58$ is significant when $p < 0.01$ [9].

Grasp vs release

All participants involved the contralateral hand more during grasping (3.48 ± 1.32s) versus releasing with the mHand (1.12 ± 0.30s) ($t(9) = 6.940,\ p < .001,\ d = 2.195$). This might be due to the fact participants did not need to touch the device/object to release its grip. With practice, most participants reduced their contralateral hand interaction time for both grasping and releasing (S2 Fig). However, it is notable that

two participants (5 and 8) saw a small increase in release interaction time between the first and last three attempts. In addition, many participants saw only limited reductions in release interaction when compared to their reductions in grasp interaction times. This might be due to a performance floor effect which is discussed in the discussion section.

S2 Fig. mHand contralateral hand involvement during grasp and release interactions. Each graph represents one participant's data for grasp and release interactions over the first or last three SHAP attempts. 'G' labels represent grasp interactions, 'R' labels represent release interactions. The numbers following 'G' or 'R' represent the series of attempts analysed. Whiskers represent the standard error.

Direct vs indirect

There is a general trend towards more direct (3.07 ± 1.48s) contralateral interaction compared to indirect (1.54 ± 0.34s) interaction ($t(9) = 3.362,\ p = 0.008,\ d = 1.063$). This differs from the SGH where the use of direct and indirect interactions varied widely between participants [4]. With practice, this trend either continued or the amount of time spent in each levelled out (S3 Fig). Similar to the other measures, participants generally reduced their contralateral hand use for both direct and indirect interactions, however, it is worth noting that two participants (2 and 6) increased their indirect interactions between the first and last three attempts.

S3 Fig. mHand contralateral hand involvement during direct and indirect interactions. Each graph represents one participant's data for direct and indirect interactions over the first or last three SHAP attempts. 'D' labels represent direct interactions and 'I' labels represent indirect interactions. The numbers following 'D' or 'I' represent the series of attempts analysed. Whiskers represent the standard error.

S1 File. Raw Data. Spreadsheet of contralateral hand interaction data for the mHand and SHAP scores for the mHand and SGH.

S2 Table. **Summary of grasps used per task.**

Task	Expected grasp	Tip	Lateral	Tripod	Spherical	Power	Extension	Other observations
**1: Light spherical**	**Spherical**	6	0	0	54	0	0	
**2: Light tripod**	**Tripod**	11.5	2	46.5	0	0	0	
**3: Light power**	**Power**	2	0	2	6	47	3	
**4: Light lateral**	**Lateral**	3.5	12	43.5	0	0	0	One attempt a participant slotted the handle between the index and middle finger.
**5: Light tip**	**Tip**	51	0	7	0	0	2	
**6: Light extension**	**Extension**	10	0	3	0	0	47	
**8: Heavy tripod**	**Tripod**	7	0	53	0	0	0	
**9: Heavy power**	**Power**	1.5	0	1	5.5	50	2	
**10: Heavy lateral**	**Lateral**	0	13	47	0	0	0	
**11: Heavy tip**	**Tip**	54	2	4	0	0	0	
**12: Heavy extension**	**Extension**	10	3	1	0	0	46	
**13: Pick up coins**	**Tip/ tripod**	56.5	0	2.5	0	0	0	1 DNF
**14: Button board**	**Tripod**	5.5	0	53.5	0	0	0	1 DNF
**16: Page turning**	**Tripod/ extension**	5	3	52	0	0	0	
**17: jar lid**	**Spherical/ power**	0	0	0	53	7	0	
**18: Glass jug pouring**	**Lateral**	0	19.5	1.5	0	38	0	1 DNF
**19: Carton pouring**	**Power**	0	0	1	0	59	0	
**22: Lifting a tray**	**Lateral/ extension**	0	6	6	0	0	48	
**23: Rotate a key**	**Lateral**	8	10	40	0	0	0	2 DNFs
**24: Open/ close zip**	**Lateral/ tip**	45	0	6	0	0	0	2 DNFs. Six attempts participants put zip between index and middle fingers. One time began with a tip grip then switched to pushing zipper head itself.
**25: Rotate a screw**	**Power**	0	0	6	1.5	44	8.5	
**26: Door handle**	**Power**	0	0	0	0	60	0	
**Sum of Attempts:**		**278**	**71**	**377**	**120**	**305.5**	**156.5**	7 DNFs, 8 alternate methods used

The 'Expected grasp' column indicates what grip SHAP expects to be used. The number in each column under the six grip types indicates the number of times that grip was used to complete the task (note attempts participants failed are not counted). If a participant used a combination of two types of grip this was counted as 0.5 per grip type. The last row shows the sum of each grip type used. Notes about alternate methods and DNFs are noted in the 'other observations' column. (DNF = did not finish)
