# Supplementary figures and images for: Correction: A quantitative comparison between the mHand Adapt passive adjustable hand prosthesis and its predecessor, the Delft Self-Grasping Hand
Source: PLoS One. 2025 Apr 4;20(4):e0322238. doi: 10.1371/journal.pone.0322238 (PMC11970660; doi:10.1371/journal.pone.0322238)

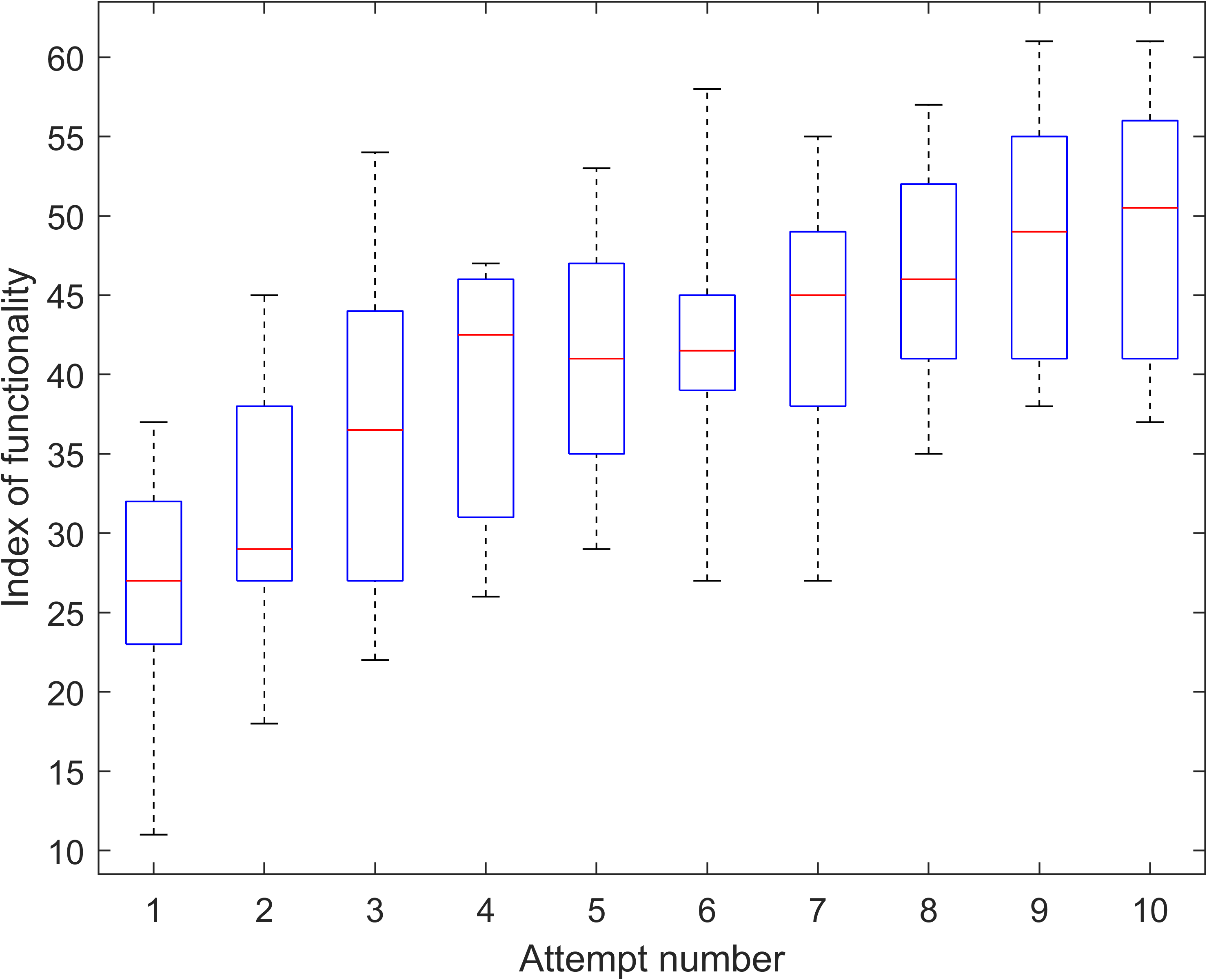

Supplement: S1 Fig — Data from all participants were combined into this box plot to show scores for each successive attempt. This figure analyses the complete mHand data set. For each attempt, the line within the box represents the median, the upper and lower edges of the box represent the upper and lower quartiles respectively, and the ends of the whiskers are the maximum and minimum values. (TIF) [file pone.0322238.s001.tif]
